# Crystal Shred Gold

**Published:** 1866-10

**Authors:** 


### CRYSTAL SHRED GOLD.

*Manufactured by* E. Lamm; *For sale by* Samuel S. White.

I have seen two specimens of this gold—have tried one. It works well—better and with more facility than the New York preparation. Its introduction will prove a professional blessing I would buy it and use it all the time, were it not that we prepare, for our own use, an article still better. I greatly prefer it to foil.

A friend says he got a specimen that crumbles badly. It isn't his fault, for he knows how to use it. I hope that piece was an accident. W.

